# MakC and MakD are two proteins associated with a tripartite toxin of *Vibrio cholerae*

**DOI:** 10.3389/fmicb.2024.1457850

**Published:** 2024-10-03

**Authors:** Nandita Bodra, Eric Toh, Aftab Nadeem, Sun Nyunt Wai, Karina Persson

**Affiliations:** ^1^Department of Chemistry, Umeå University, Umeå, Sweden; ^2^Umeå Center for Microbial Research (UCMR), Umeå University, Umeå, Sweden; ^3^Department of Molecular Biology, Umeå University, Umeå, Sweden; ^4^The Laboratory for Molecular Infection Medicine Sweden (MIMS), Umeå University, Umeå, Sweden

**Keywords:** secretion, toxin, liposome, crystal structure, *Vibrio cholerae*

## Abstract

Pathogenic serotypes of *Vibrio cholerae*, transmitted through contaminated water and food, are responsible for outbreaks of cholera, an acute diarrheal disease. While the cholera toxin is the primary virulence factor, *V. cholerae* also expresses other virulence factors, such as the tripartite toxin MakABE that is secreted via the bacterial flagellum. These three proteins are co-expressed with two accessory proteins, MakC and MakD, whose functions remain unknown. Here, we present the crystal structures of MakC and MakD, revealing that they are similar in both sequence and structure but lack other close structural relatives. Our study further investigates the roles of MakC and MakD, focusing on their impact on the expression and secretion of the components of the MakABE tripartite toxin. Through deletion mutant analysis, we found that individual deletions of *makC* or *makD* do not significantly affect MakA expression or secretion. However, the deletion of both *makC* and *makD* impairs the expression of MakB, which is directly downstream, and decreases the expression of MakE, which is separated from *makCD* by two genes. Conversely, MakA, encoded by the *makA* gene located between *makB* and *makE,* is expressed normally but its secretion is impaired. Additionally, our findings indicate that MakC, in contrast to MakD, exhibits strong interactions with other proteins. Furthermore, both MakC and MakD were observed to be localized within the cytosol of the bacterial cell. This study provides new insights into the regulatory mechanisms affecting the Mak protein family in *V. cholerae* and highlights the complex interplay between gene proximity and protein expression.

## 1 Introduction

*Vibrio cholerae*, a Gram-negative bacterium with a distinctive comma shape, presents a considerable challenge to public health and aquatic ecosystems (Clemens et al., 2017). Its pathogenic characteristics often lead to cholera outbreaks, causing significant health crises and economic impacts (Jutla et al., 2017). There has been six recorded cholera pandemics since the early 19th century and we are currently experiencing the seventh pandemic, which has persisted since 1961 (Karaolis et al., 1994). While *V. cholera* naturally resides in environmental waters, human infection primarily occurs through the consumption of contaminated food or water. Cholera disease is mainly caused by a limited number of serogroups that express cholera toxin (CT) and the toxin co-regulated pilus (TCP) (Herrington et al., 1988; Lee et al., 2023). The TCP facilitate bacterial colonization in the intestine (Kirn et al., 2000) and the toxin induces severe disruption of intestinal cell function, resulting in profuse watery diarrhea. Extensive research has investigated the pathogenic mechanisms and the regulation of virulence factors of *V. cholerae* in human hosts. However, there is increasing interest in understanding how *V. cholerae* manages to survive and thrive in environmental settings, especially in the presence of predatory organisms. The bacterium’s environmental resilience involves a range of adaptive strategies, including the formation of biofilms, quorum sensing, and genetic variations that enhance survival against predators and fluctuating environmental conditions (Zhu et al., 2002; Alam et al., 2006; Safa et al., 2010). These adaptations not only facilitate its persistence in the environment but also play a crucial role in the bacterium’s ability to re-enter human populations, thus perpetuating the cycle of infection.

Our recent studies identified a cytotoxin, motility associated killing factor A (MakA), important for the cytotoxic effects of *V. cholerae* on both *Caenorhabditis elegans* and zebrafish. Furthermore, we uncovered its unique secretion pathway via the flagellum, a mechanism not previously described in *V. cholerae* (Dongre et al., 2018). Our further investigation demonstrated that MakA can assemble with MakB and MakE to form a tripartite *α*-pore-forming toxin complex responsible for cell toxicity (Nadeem et al., 2021). Analyzing the crystal structures of MakA, MakB, and MakE revealed their resemblance to the ClyA family of bacterial pore-forming toxins (Dongre et al., 2018; Nadeem et al., 2021).

**Figure 1:**
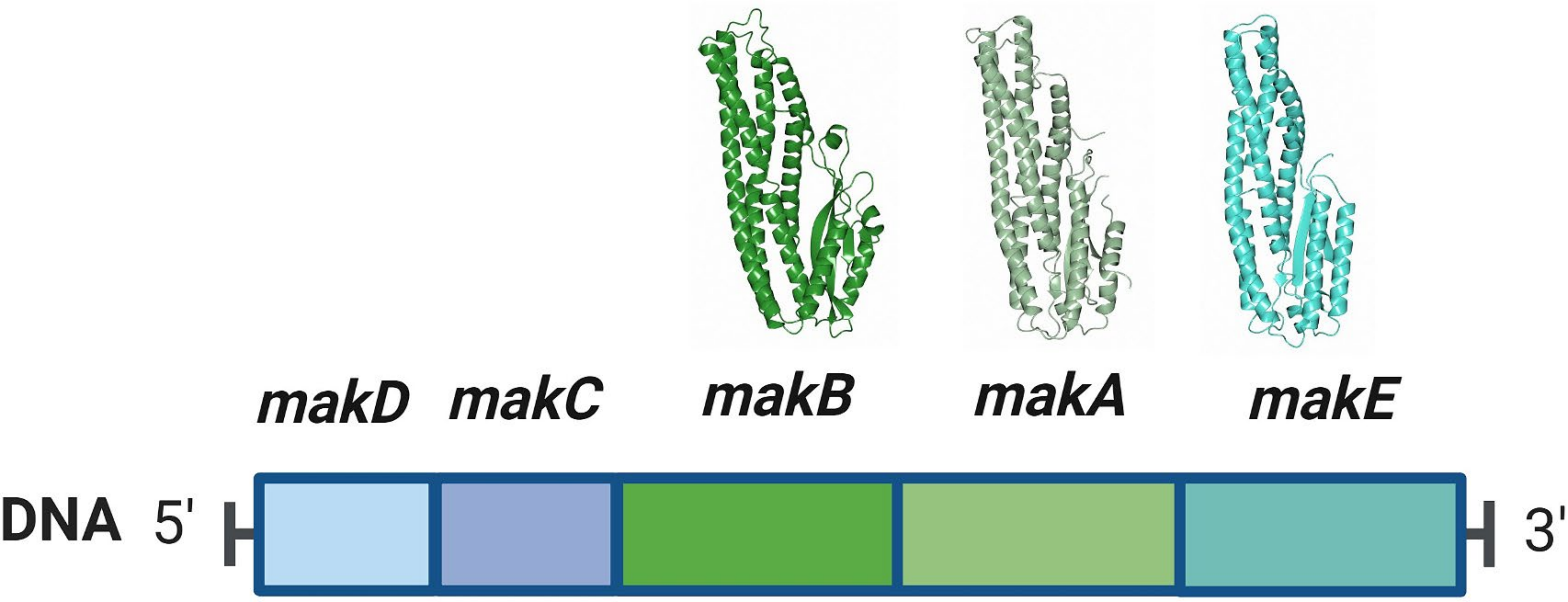
The *makDCBAE* operon of *Vibrio cholerae*. The encoded proteins MakA, MakB, and MakE are secreted via the bacterial flagellum and form a tripartite toxin complex that will bind the host membrane. In order to penetrate the membrane, they first have to undergo a conformational change. MakC and MakD are small accessory proteins with unknown functions and are not secreted. The crystal structures of MakA (pdb: 6EZV), MakB (pdb: 6T8D), and MakE (pdb: 6TAO) are shown above their respective genes.

MakA, MakB, and MakE are encoded from the *makDCBAE* operon (Figure 1). This operon is under the control of HapR, a quorum sensing-regulated transcriptional factor that also downregulates CT production (Tsou et al., 2009). Additionally, it was found that the expression of the *makDCBAE* operon is dependent on the bacterial growth phase, being activated in the stationary phase by the transcriptional regulator RpoS (Zhang et al., 2019). This suggests that Mak proteins are not predominantly expressed during the acute phases of cholera infection but are more likely to be synthesized in natural aquatic environments, where the challenges of nutrient scarcity and predation significantly influence bacterial fitness and survival.

Bioinformatic analyses revealed that the *makDCBAE* operon exists as a genomic island in the vast majority of *V. cholerae* and *Vibrio anguillarum* sequenced genomes. We propose that many pathogenic *Vibrionaceae* strains possess a previously unknown potential to generate the tripartite Mak cytolytic toxin, which may contribute to *Vibrionaceae* fitness and virulence potential in a variety of host environments and organisms (Nadeem et al., 2021). The genes *makC* and *makD* are positioned immediately upstream of the gene encoding MakB. Our earlier studies have shown that mutations in the *makC* and *makD* genes have minimal impact on virulence against *C. elegans*, suggesting a specialized yet unknown role in virulence (Nadeem et al., 2021).

The specific functions of MakC and MakD proteins in the pathogenesis and environmental resilience of *V. cholerae* remain uncharacterized. These proteins are not secreted via the flagellum in the manner of other Mak proteins (Nadeem et al., 2021), suggesting a divergent role in the bacterium’s life cycle. The elucidation of the roles of MakC and MakD in *V. cholerae*’s pathogenicity and environmental adaptation remains an open area for future research. Addressing this knowledge gap could provide deeper insights into the complex host-pathogen interactions and potentially reveal new targets for therapeutic intervention or strategies to mitigate *V. cholerae* infections.

In this study, we present the structural characterizations of the MakC and MakD proteins. Additionally, we explore the effect of these proteins on other members of the Mak protein family and their behavior in the presence of liposomes.

## 2 Materials and methods

### 2.1 Expression and purification of MakC and MakD

The *makC* and *makD* genes (Uniprot: Q9KL66 and Q9KL67) were PCR amplified from genomic DNA from the *V. cholera* strain A1552 and cloned into pET-His1a, in-frame with a cleavable linker and histidine affinity tag with the sequence MKHHHHHHPMSDYDIPTTENLYFQGA (strains and primer sequences are presented in Supplementary Table 1). The purified plasmids were transformed into *E. coli* BL21 (DE3) and precultures were prepared using LB broth supplemented with 50 μg/mL kanamycin. The precultures were used to inoculate larger cultures in which protein expression was induced with 0.5 mM isopropyl 1-thio-*β*-d-galactopyranoside (IPTG) at OD_600_ ~ 0.6 followed by growth for 5 h at 25°C. The cells were harvested by centrifugation, and the pellets were stored at-80°C until further use.

MakC and MakD were purified using similar protocols. In short, cell pellets were resuspended in lysis buffer (50 mM Tris–HCl pH 7.6, 0.3 M NaCl, and 10 mM imidazole) containing 1% triton X-100 and sonicated on ice. The lysate was centrifuged for 30 min at 60,000 × *g* and the supernatant was collected. The supernatant was incubated with His60 Superflow resin (Takara Bio) for batch purification. The beads were transferred to a column and washed with wash buffer (50 mM Tris–HCl pH 7.6, 0.3 M NaCl, and 30 mM imidazole), and next the protein was eluted with elution buffer (50 mM Tris–HCl pH 7.6, 0.3 M NaCl, and 0.3 M imidazole). Furthermore, dialysis and histidine tag removal were simultaneously performed by incubating the protein with 1% (w/w) TEV protease overnight at 4°C in 50 mM Tris–HCl pH 7.6, and 0.2 M NaCl. The dialyzed protein solution was again passed over the His60 Superflow resin, and the flow-through was collected and concentrated using 10 kDa amicon centrifugal units (Millipore). The cleaved protein was further purified on a HiLoad 16/600 Superdex 200 prep grade column (Cytiva) equilibrated with 20 mM Tris–HCl pH 7.6 and 0.2 M NaCl. The eluted fractions were analyzed on SDS-PAGE, and the protein was concentrated. Selenomethionine (SeMet)-labeled MakC and MakD were produced by growing the bacterial cultures in M9 media supplemented with glucose at 37°C. At an OD_600_ of ~0.4, 100 mg/L each of lysine, threonine, phenylalanine, and 50 mg/L each of leucine, isoleucine, valine, proline, and SeMet were added, and expression was induced with 0.5 mM IPTG and grown at 20°C overnight (Doublie, 1997). The SeMet labeled proteins were purified as the native proteins.

### 2.2 Crystallization and structure determination

All crystallization screenings were performed by the sitting-drop vapor-diffusion method in 96-well MRC-crystallization plates (Molecular Dimensions) using a Mosquito pipetting robot (TTP Labtech). Commercial screens from Molecular Dimensions and Hampton Research were used for the initial screening.

MakC-SeMet was concentrated to 15 mg/mL in 20 mM Tris, pH 7.6, 0.2 M NaCl. Crystals grew in 0.2 M potassium sodium tartrate, 0.1 M sodium citrate, pH 5.6, and 2 M ammonium sulfate (200 nL protein:100 nL crystallization solution) and were cryoprotected in mother liquor supplemented with 20% (v/v) glycerol before vitrification in liquid nitrogen. MakD was concentrated to 10 mg/mL in 20 mM Tris pH 7.6. Native MakD was crystallized in 0.1 M Tris–HCl pH 8.0 and 60% (v/v) polypropylene glycol; MakD-SeMet crystals were grown in 50 mM NaH_2_PO_4_, 24% (w/v) PEG 8000. For native MakD no cryo-protectant was used, whereas MakD-SeMet crystals were cryoprotected with 20% (v/v) PEG400.

MakC data were collected remotely on beamline ID30B at the European Synchrotron Radiation Facility (ESRF), Grenoble, France on an EIGER 4 M detector. Images were integrated and scaled using AutoPROC (Evans, 2006; Evans and Murshudov, 2013; Kabsch, 2010; Vonrhein et al., 2011) and STARANISO, achieving a resolution of 2.0 Å. The structure was determined by Single Anomalous Dispersion (SAD) phasing using CRANK2 (Skubak et al., 2018; Skubak and Pannu, 2013), and the initial model was built with Buccaneer of CCP4i (Martinez-Ripoll and Albert, 2023). Further rounds of model building and refinement were carried out using COOT (Emsley et al., 2010) and Refmac5 (Murshudov et al., 2011).

Native MakD data were collected at beamline ID23-1 on an EIGER 4 M detector and SAD MakD data on a PILATUS 6MF detector at beamline ID29 (ESRF). Diffraction images were processed with XDS (Kabsch, 2010) and scaled with Aimless (Evans and Murshudov, 2013) from the CCP4 program suite (Winn et al., 2011). The structure of SeMet-labeled MakD was solved using phenix autosolve (Afonine et al., 2012). The crystals of native MakD grew in a different crystal form than the SeMet-labeled protein, and hence the structure was obtained by molecular replacement using Phaser (McCoy, 2007) with the SeMet structure as a search model. The native structure was refined using phenix.refine (Afonine et al., 2012) and built using rounds of manual building in COOT (Emsley et al., 2010). For the refinement of MakD, translational-libration-screw refinement was used, treating each molecule as an individual TLS group (Winn et al., 2001). All data processing and refinement statistics are presented in Supplementary Table 2. Figures of protein structures are prepared with CCP4mg (McNicholas et al., 2011). The protein sequences were aligned with T-Coffee (Floden et al., 2016) and visualized with Espript3 (Robert and Gouet, 2014).

### 2.3 Analysis of secreted proteins by SDS-PAGE and immunoblotting

Analysis of protein secretion from *V. cholerae* was performed as published previously (Dongre et al., 2018). Briefly, bacterial cultures were centrifuged to separate cells from the supernatant. The cell pellets were resuspended in 1 × SDS buffer and boiled. Supernatants were filtered through a 0.45 μm PVDF filter (Millipore, United States). Next, the proteins in the supernatant were precipitated with 10% (w/v) Trichloroacetic acid (TCA) and centrifuged for 15 min at 15,000 *g*. The pelleted TCA-precipitated proteins were resuspended in 1 x SDS buffer and separated by SDS-PAGE, followed by transfer to a nitrocellulose filter. The membrane was incubated with specific antisera, including anti-MakA (1:5,000 dilution) anti-MakB (1:5,000 dilution), anti-MakE (1:5,000 dilution), anti-MakC (1:5,000 dilution) anti-MakD (1:5,000 dilution) (Nadeem et al., 2021) (produced by GeneCust), anti-HapA (1:5,000 dilution) (Vaitkevicius et al., 2006) and anti-CRP (1:3,000 dilution) (Ishikawa et al., 2009), HPR-conjugated goat-antirabbit IgG (Agrisera, Sweden) was used as the secondary antibody. Detection was performed with Clarity Western ECL substrate (BioRad).

### 2.4 *In vivo* crosslinking of MakC and MakD in *Vibrio cholerae*

For the *in vivo* glutaraldehyde crosslinking experiment, *V*. *cholerae* A1552 WT were grown in LB at 37°C until OD_600_ of 2.0 and bacterial culture samples were collected at this time point. The remaining bacterial culture samples were treated with 0.25% glutaraldehyde and incubated at 37°C while shaking for another 10 min. Subsequently, the non-treated and treated bacterial cells were collected by centrifugation at 10,000 × *g* for 10 min at 4°C. The cell pellets were resuspended in 80 μL of 1 × SDS sample buffer and boiled for 10 min to obtain whole cell lysates. The protein samples were resolved by SDS-PAGE and processed for Western blotting using anti-MakC (1:5,000 dilution) and anti-MakD antisera (1:5,000 dilution).

### 2.5 Liposome preparation

Lipids from the wild type *V. cholerae* A1552 strain were extracted by the Folch method (Folch et al., 1957). Briefly, the *V. cholerae* A1552 strain was cultured overnight at 37°C in LB medium. Lipids were then extracted using chloroform. After the chloroform was removed with a stream of nitrogen, the resulting lipid film was obtained and dried. The lipid film (5 or 10 mg/mL) was hydrated with 20 mM citrate, 50 mM NaCl, pH 4.5 at 37°C and the solution was extruded over polycarbonate membranes with a 0.1 μm pore size using the Avant Mini-extruder (Avanti Polar lipids, Alabaster, AL). The liposomes were stored at 4°C until use.

### 2.6 Liposome pulldown assay

The liposome extracts prepared from the *V. cholerae* A1552 strain were incubated with MakC (5 μM) or MakD (5 μM) in 120 mM citrate buffer, pH 6.5 at 37°C for 2 h. The reaction mixture was then cross-linked with 0.05% glutaraldehyde for 10 min at 37°C, followed by the addition of 200 mM Tris pH 6.8, to stop the reaction. Subsequently, the sample was centrifuged at 21,500 × *g* for 30 min. The pellet containing complexes bound to liposomes was washed twice with buffer, separated on SDS-PAGE, and then subjected to Western blot analysis using MakC and MakD specific antisera.

### 2.7 Confocal microscopy

The freshly prepared *V. cholerae* A1552 strain was grown in an 18-well chamber slide under stationary conditions at 37°C in LB for 4 h, followed by staining with lipophilic membrane dye, FM 4-64FX for 10 min at room temperature. The unbound membrane dye was washed off, followed by fixation of the bacteria with 4% paraformaldehyde. The bacteria adhered to the glass surface after fixation were permeabilized with Triton X-100 (0.1%), followed by incubation with antisera against MakC and MakD proteins [1:100 dilution in 5% Fetal Calf Serum (FCS)/PBS], overnight at 4°C. The unbound antibodies were washed off with PBS (three times), and the proteins MakC and MakD were detected using Alexa488-conjugated secondary antibodies (1:200 dilution in 5% FCS/PBS) at room temperature for 1 h. After washing with PBS (three times), the proteins MakC and MakD were visualized with a Leica SP8 confocal microscope (Leica Microsystems) equipped with an HC PL APO 63×/1.40 oil immersion lens. Images were analyzed and processed in ImageJ (NIH). Fluorescence intensity profiles for the selected regions of interest were generated using the plot profile command in ImageJ (Schindelin et al., 2012).

## 3 Results

### 3.1 Overall structure of MakC and MakD

The proteins MakC and MakD are encoded by the *mak* operon (Figure 1). To determine the crystal structure of MakC, a construct encoding the full length MakC, with an N-terminal cleavable histidine tag, was designed and expressed in *E. coli*. The calculated molecular weight of the protein, without tag, was 14.4 kDa which was confirmed by SDS-PAGE (Supplementary Figure 1). After the removal of the histidine tag, several crystallization screens were set up, but diffraction quality crystals were not obtained. Consequently, SeMet labeled MakC was prepared, yielding needle-like crystals. These needles were used as seeds to optimize crystal growth, resulting in long, thin crystals. The crystals, which belonged to space group C222_1_ and contained one molecule in the asymmetric unit, diffracted to 2.0 Å. The structure of MakC was determined by SAD phasing on SeMet labeled protein. The final model was refined to R and Rfree of 0.18 and 0.20, respectively.

MakD, with a calculated molecular weight of 14.0 kDa was similarly expressed with a hexahistidine tag (Supplementary Figure 1). Crystals of SeMet-MakD, without tag, were obtained in several conditions and the crystal structure was solved using SAD phasing. This model was used to determine the structure of the native protein, which was refined to 2.0 Å. The native protein crystallized in space group P2_1_2_1_2_1_ with four molecules in the asymmetric unit, forming two identical dimers. The final model was refined to R and Rfree of 0.18 and 0.23, respectively. Processing and refinement statistics are given in Supplementary Table 2.

**Figure 2:**
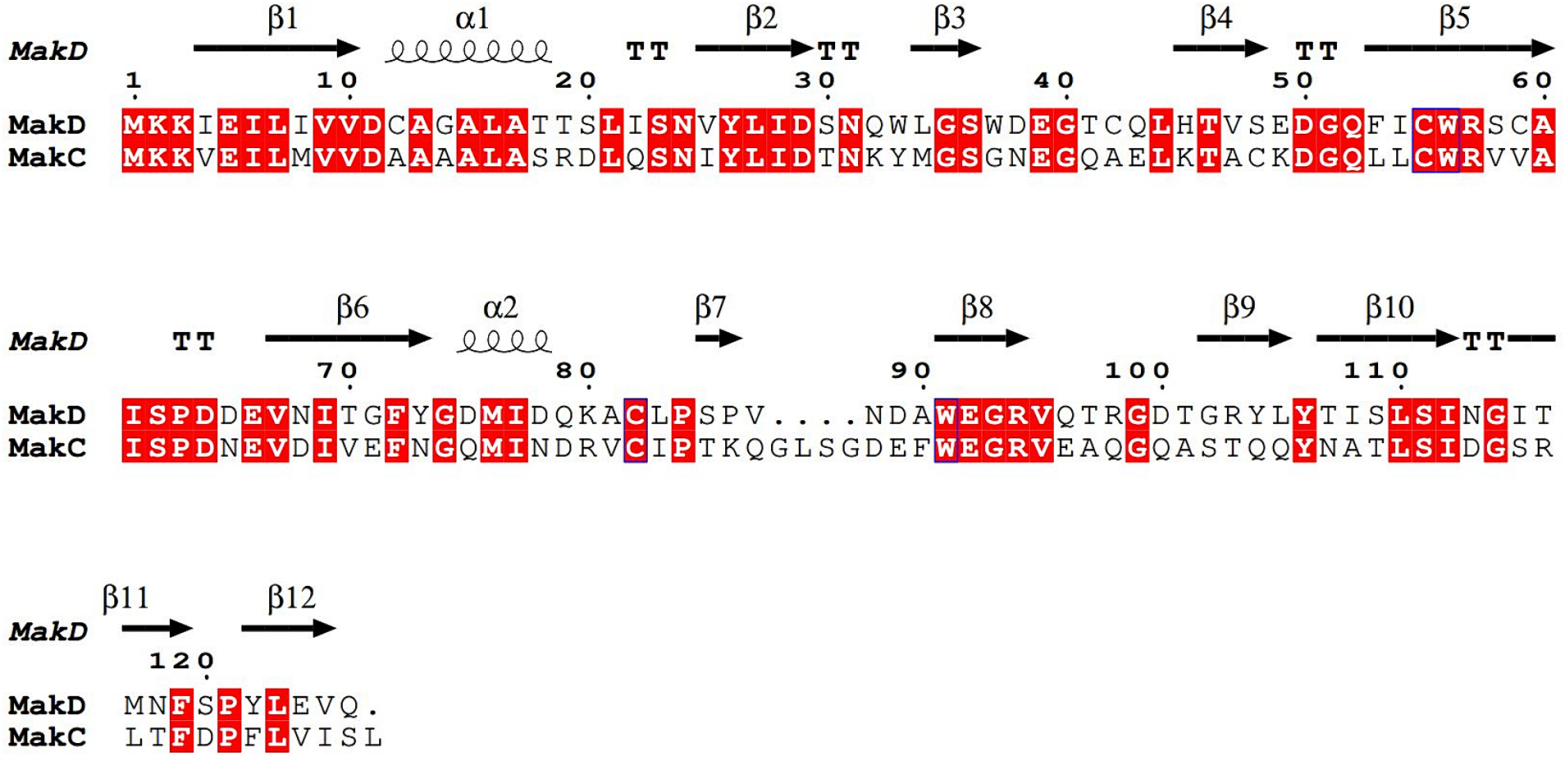
Sequence alignment of MakC and MakD. The protein sequences of MakC and MakD were aligned with TCoffee and visualized with Espript3. The sequence identity is 48%. The secondary structure of MakD is indicated above the sequences.

**Figure 3:**
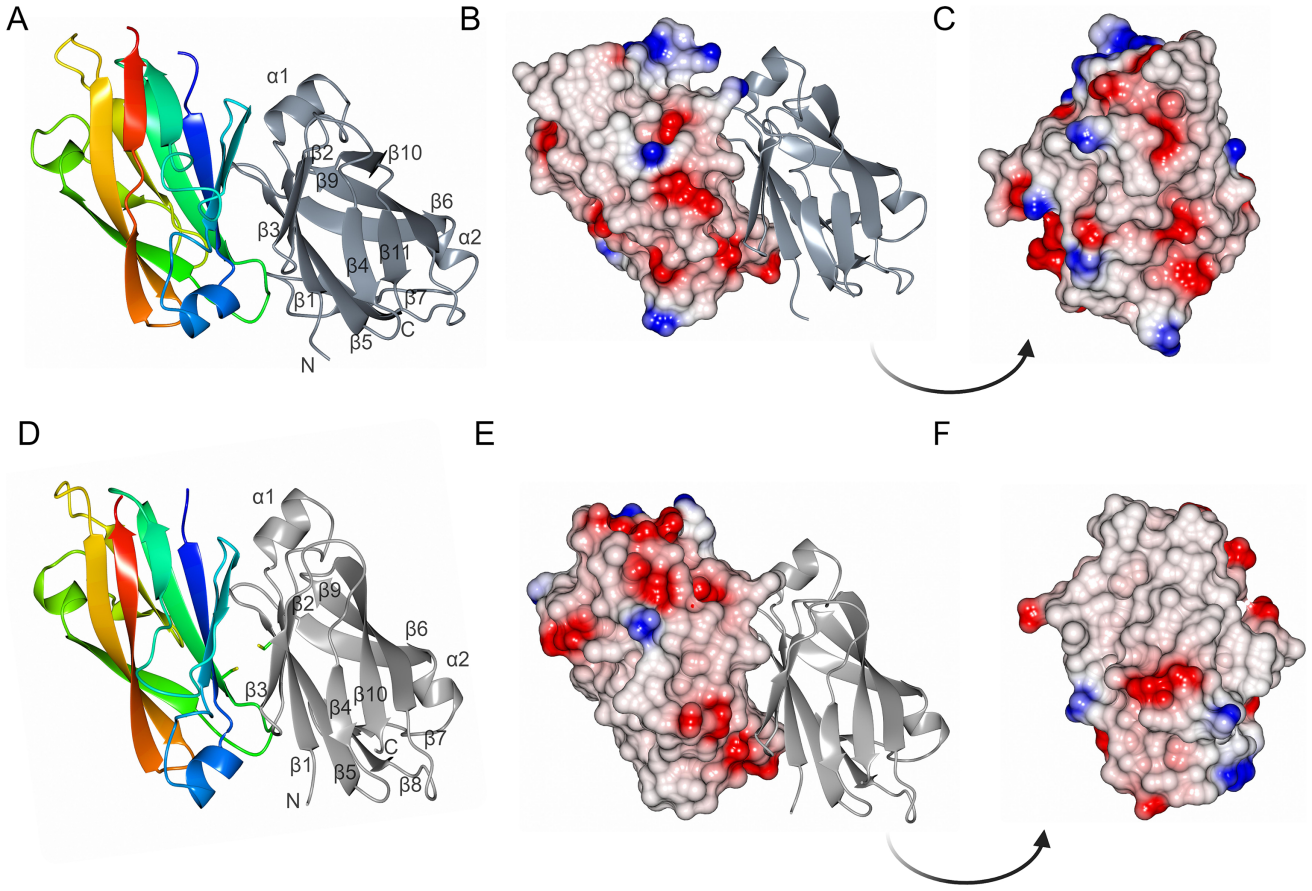
The overall structures of MakC and MakD. **(A)** The overall structure of MakC is depicted as a ribbon model colored from blue (N-terminus) to red (C-terminus). The chain of the crystallographic dimer is depicted in gray. **(B,C)** MakC depicted as an electrostatic potential map. **(D)** The overall structure of MakD. One of the chains is colored from blue to red, and the other is gray. The cysteines that may form a disulfide bond are shown as stick models. The asymmetric unit contains four chains, but only two are shown here for clarity. **(E,F)** MakD represented as electrostatic surface.

MakC and MakD comprise 131 and 126 amino acids, respectively, with a sequence identity of 48% (Robert and Gouet, 2014; Madeira et al., 2022) (Figure 2). The structures are very similar with a root mean square deviation (RMSD) of 0.45 Å calculated on 126 Cα-atoms (Supplementary Figure 2A). Both feature a central *β*-sandwich, one sheet comprising six β-strands, and one with four β-strands. Both β-sheets consist of a combination of parallel and anti-parallel strands. In addition, there is one helix located between β1 and β2, and a small helix between β6 and β7 (Figures 3A,D).

Both MakC and MakD form dimers in their respective crystal structure. In MakD, two dimers were present in the asymmetric unit, whereas the MakC dimer is formed by crystallographic symmetry. Both the MakC and MakD homodimers are formed by the interaction between identical surfaces in each subunit, head-to-tail, (β5123 pack anti-parallel to the equivalent strands, β5’1′2′3′ of the other subunit). As calculated by PISA (Krissinel and Henrick, 2007), each MakC subunit buries approximately 1,050 Å^2^ and each subunit of MakD 943 Å^2^. This represents about 15% of the total surface of the respective protein. The MakD dimer is possibly stabilized by a disulfide bond between Cys59 on β5 in each of the subunits. In MakC, the residue in the equivalent position is a valine, and there are no other cysteines in MakC that could potentially form disulfide bonds in the dimer-dimer interface. Electrostatic potential surfaces reveal that sheet 2 of MakD is predominantly non-polar, whereas some of the loops connecting the strands are more charged, for instance, the loop between β5 and β6. The equivalent β-sheet of MakC is less hydrophobic based on the electrostatic potential map, and the polar residues are more evenly distributed (Figure 3).

Since MakC and MakD can form homodimers, we used Alphafold2 to investigate whether they also can form heterodimers (Mirdita et al., 2022). The generated MakCMakD dimer was predicted to have the same dimer-dimer interface and looked very similar to the two MakC and MakD homodimers (Supplementary Figure 2B). It is important to note that interactions are only predictions hence, further evaluation is needed.

### 3.2 Comparative structural analysis of MakC and MakD

A DALI search (Holm et al., 2023) performed on the structures of MakC and MakD yielded hits with relatively low *Z*-scores, indicating a lack of highly similar structures in the Protein Data Bank. The search identified three hits with *Z*-scores higher than 10. These matches are uncharacterized proteins from *Burkholderia cenocepacia* (pdb:4lzk), *Chitinophaga pinensis* (pdb:4q52), and *Burkholderia thailandensis* (pdb:4pib), displaying RMSDs of 2.0, 2.3, and 2.2 Å, respectively (Supplementary Figure 3). The lack of described functions for these proteins is intriguing, prompting further investigation into the potential roles of MakC and MakD in contributing to the environmental adaptability of *V. cholerae*.

To further explore structural homologs, FoldSeek (van Kempen et al., 2024) searches were performed using MakC and MakD structures against the PDB100 database, with *V. cholerae* as a taxonomic filter. This investigation revealed a common hit for MakC and MakD—the C-terminal domain of the VesB protease (Gadwal et al., 2014). The function of the VesB C-terminal domain is unknown, but it was discussed whether the domain involved stabilization of the protease domain, secretion via the type II secretion system, or binding to the bacterial surface (Gadwal et al., 2014). The FoldSeek analysis without taxonomic filtering against the AFDB-Uniprot, AFDB-Swissprot, and CATH databases revealed many structural homologs, but all with high E-values which indicates a low confidence level.

### 3.3 Intracellular localization of MakC and MakD and interdependency of Mak proteins

**Figure 4:**
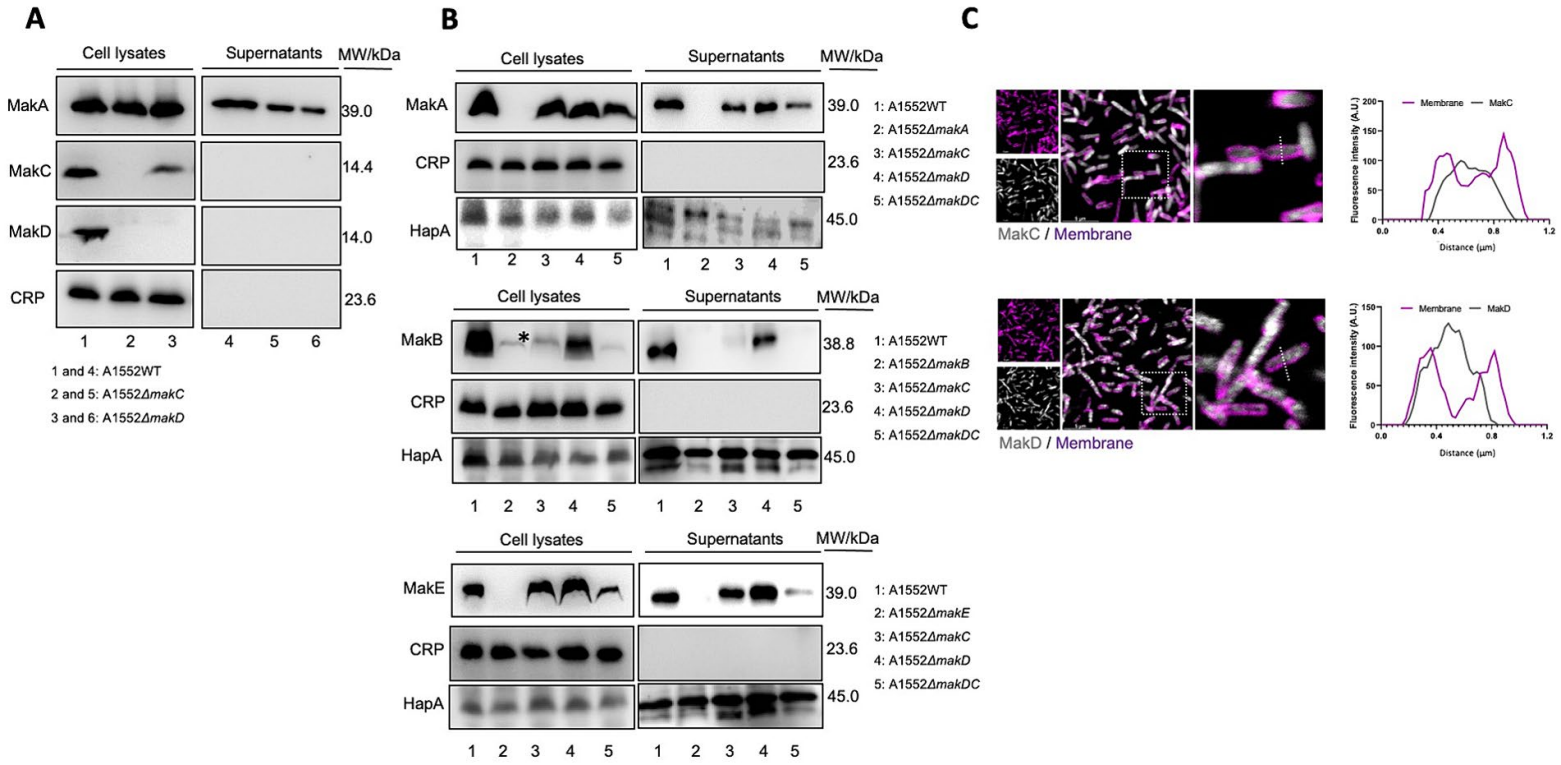
Western immunoblot of Mak protein expression and secretion from *Vibrio cholerae* O1 El Tor strain A1552. **(A)** Samples of bacterial cell lysates (lanes 1–3) and culture supernatants (lanes 4–6) were subjected to immunodetection with antisera raised against MakA, MakC, and MakD, respectively. **(B)** Western blot analysis of MakA (panel 1), MakB (panel 2), and MakE (panel 3) in whole cell lysates or secreted to the supernatant. The study was done on wild-type *V. cholerae* A1552, *ΔmakA*, *ΔmakB*, *ΔmakE*, and *ΔmakDC.* Cyclic AMP receptor protein (CRP), a cytoplasmic non-secreted protein, was used as a control for cytoplasmic proteins, while hemagglutinin protease (HapA) served as a control for secreted proteins. The asterisk indicates an unidentified protein also detected by the MakB antiserum, as described earlier (Nadeem et al., 2021). In panels **(A,B)**, the molecular weights of the proteins are indicated. **(C)** Confocal microscopy of WT A1552 grown on a coverslip bottom glass chamber slide, stained with antisera against MakC and MakD (gray). The cell membrane of the bacterium was stained with FM 4-64FX (purple). Fluorescence intensity profiles of the corresponding image along the dotted white line were used to generate the line graphs. Scale bars = 5 μm.

We have previously demonstrated that while MakA, MakB, and MakE are secreted by the bacterium, MakC is not (Nadeem et al., 2021). In our current study, it was also revealed that MakD is only found in the bacterial cells and not in the supernatant. Hence, neither MakC nor MakD are secreted (Figure 4A, lanes 1 and 4).

We observed that deletion of the *makD* gene does not affect the expression of MakC to any greater extent (Figure 4A, lane 3). Deletion of *makC* is however completely suppressing the expression of MakD (Figure 4A, lane 2). We also investigated the effect on the remaining components, MakA, MakB, and MakE, by deleting both *makC* and *makD*. Interestingly, the expression of MakA was not affected, as the protein could be detected in the cell lysate in almost the same amount as in the wild type (Figure 4B, panel 1). The amount of MakA in the supernatant, on the other hand, decreased. The expression of MakB was abolished, and the protein cannot be detected in cell lysates (Figure 4B, panel 2). The double mutant also resulted in decreased amounts of MakE in cell lysates, however, the secretion did not appear to be affected since comparable amounts of MakE were found in both the lysate and the supernatant (Figure 4B, panel 3).

To gain insight into the cellular distribution of MakC and MakD, the *V. cholerae* A1552 strain was stained with the fixable cell membrane dye, FM 4-64FX, following fixation and staining with antisera against MakC and MakD proteins (Figure 4C). Through the analysis of confocal microscopy images, we observed that both MakC and MakD were primarily localized in the bacterium’s cytosol. Additionally, a liposome pulldown assay also confirmed that neither MakC nor MakD interact with lipids, as the proteins could only be detected in the supernatant and not in the lipid containing pellet (Supplementary Figure 4).

### 3.4 *In vivo* crosslinking reveals interactions with other proteins in the cell

**Figure 5:**
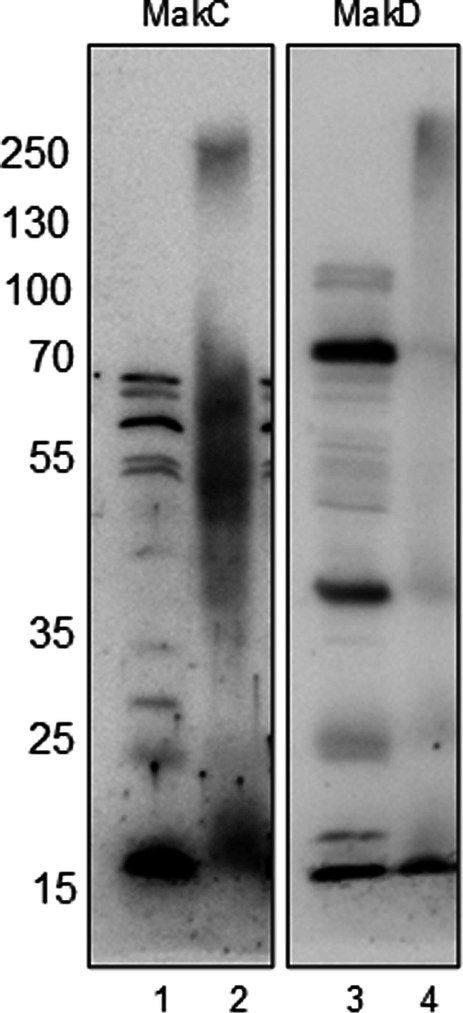
MakC and MakD are localized in the cytosol of *Vibrio cholerae*, and form oligomers upon chemical crosslinking. WT *V. cholerae* O1 El Tor strain A1552 (lanes 1 and 3) and WT A1552 treated with 0.25% Glutaraldehyde (lanes 2 and 4) were analyzed with Western immunoblotting using antisera against MakC and MakD respectively.

In our study, we performed *in vivo* crosslinking of the wild-type *V. cholerae* strain A1552 with various *mak* gene mutations to investigate potential interactions involving MakC, MakD, and other Mak proteins. Protein content was analyzed by SDS-PAGE and western blotting with specific antisera for MakC and MakD. In the analysis with MakC antiserum, smeared bands of MakC were observed and a decreasing amount of monomer protein, indicating that MakC is indeed crosslinked with other proteins within the bacterial cell (Figure 5, panel 1). Crosslinking of MakC was repeated with increasing concentrations of glutaraldehyde and performed at 10 and 30 min (Supplementary Figure 5). Interestingly, the samples analyzed with the anti-MakD antisera showed much less smearing and no apparent loss of monomer protein, suggesting that MakD engages in fewer interactions with other proteins. (Figure 5, panel 2). These results highlight intriguing differences in the crosslinking behavior of MakC and MakD, warranting further investigation. The differential crosslinking patterns may shed light on their distinct roles within the bacterial cell.

## 4 Discussion

*Vibrio cholerae* exhibits a range of virulence factors targeting host cells, of which cholera toxin and the toxin co-regulated pilus are those that have been studied in most detail. We have previously identified and characterized a tripartite toxin in *V. cholerae* composed of three structurally similar proteins: MakA, MakB, and MakE (Dongre et al., 2018; Nadeem et al., 2021). These proteins initially exist as soluble, two-domain proteins that undergo a conformational change upon contact with the host membrane, facilitated by a hinge between the domains (Nadeem et al., 2021). This change exposes transmembrane helices, allowing the proteins to assemble into a pore-forming toxin. Tripartite toxins with similar structures have been identified in other Gram-negative bacteria such as *Aeromonas hydrophila*, and *Serratia marcescens* (Wilson et al., 2019; Churchill-Angus et al., 2021), as well as in the Gram-positive *Bacillus cereus* (Sastalla et al., 2013).

MakABE are co-expressed with two accessory proteins, MakD and MakC, which are encoded by the same *makDCBAE* operon, although their functions remain unknown. Notably, no accessory proteins have been identified for other tripartite toxins, and a search of the protein structure database revealed that MakD and MakC lack close structural relatives. Additionally, the regulation and delivery of the tripartite toxins, which are produced by evolutionarily very distant bacteria, to host cells, remains an open question. Whereas MakA, MakB, and MakE are secreted via the single flagellum of *V. cholerae*, it is plausible that the other tripartite toxins utilize different transport mechanisms, potentially involving specialized secretion systems or bacterial membrane vesicles (Buchacher et al., 2023). It is also important to note the significant differences in the flagellar architecture of bacteria that are relatively closely related. For example, *V. cholerae* and *E. coli,* both *γ*-proteobacteria, exhibit distinct flagellar structures. These differences likely reflect adaptations to their respective environments: *V. cholerae* possesses a sheathed single polar flagellum driven by a sodium gradient (Kojima et al., 1999; McCarter, 2001), whereas *E. coli* expresses multiple flagella driven by a proton gradient (Blair, 2003; Lo et al., 2013).

The exclusive presence of the toxin-associated proteins MakC and MakD in *V. cholerae* may be linked to the unique composition of its flagellum and the specific requirement to secrete these toxin components (Zhu et al., 2017). It is plausible to speculate that MakC and MakD might function to switch the flagellum from a motility apparatus to a secretion system, possibly through direct or indirect interactions with the proteins of the flagellar C-ring located on the cytoplasmic side (Zhu et al., 2017). Alternatively, since MakA, MakB and MakE must adapt an elongated form, or unfold, in order to be able to pass through the narrow flagellar channel, similar to how proteins are transported through the type-3 injectisome (Radics et al., 2014), MakC and MakD could function to stabilize these toxins before they enter the flagellum. Further investigations are needed to determine whether MakC and MakD play regulatory roles in stabilization or in controlling the transition between the motile and secretory functions of the flagellum.

The immunoblot analysis from a previous study (Dongre et al., 2018) revealed that the genes within the *mak* operon are subject to regulation by the gene located directly upstream, indicating a transcriptional polar effect. Specifically, the deletion of *makD* results in reduced production of MakC, the deletion of *makC* diminishes the levels of MakB, and the deletion of *makB* completely abolishes the production of MakA. Notably, the production of MakE remains unaffected by the deletion of the upstream gene *makA*. In the present study, we examined the effects of deleting *makD* and *makC,* as well as the double mutant Δ*makDC.* As expected, only minimal levels of MakB are produced in the *ΔmakCD* mutant. Interestingly, while MakA is expressed at nearly wild-type levels in the double mutant, the amount of secreted MakA is significantly reduced, suggesting that MakB is essential for efficient MakA secretion. The expression and secretion of MakE are also reduced in the Δ*makDC* mutant, although the changes in MakE protein levels are less pronounced compared to the substantial reductions observed for MakB and the secreted form of MakA. Notably, also the deletion of a downstream gene can interfere with protein production, as evidenced by the complete loss of MakD expression in the Δ*makC* mutant. The regulation of the genes and gene products within the *makDCBAE* operon presents intriguing complexities that warrant investigation at both the RNA and protein levels.

*Vibrio cholerae* has a complex life cycle and has continuously undergone evolutionary adaptations to enhance its environmental fitness. This pathogen is capable of surviving for extended periods in coastal and estuarine environments without any contact with a human host. To persist in these fluctuating environments, *V. cholerae* has developed a range of protective mechanisms that allow it to withstand variations in temperature, nutrient availability, salinity, and predation. These adaptive characteristics, beyond the well-characterized virulence factors necessary for human infection, are critical for the bacterium’s survival in the diverse and often hostile conditions (Conner et al., 2016). One such adaptive mechanism is biofilm formation, where the bacterium protects itself with an extracellular matrix.

While the cholera toxin and the toxin co-regulated pilus are crucial for human infection, we hypothesize that the Mak proteins play a significant role in *V. cholerae*’s survival strategy, particularly in defending against predation by other aquatic organisms. The transcription of the *mak* operon is regulated by the quorum sensing-controlled transcription factor HapR, which also represses biofilm formation (Tsou et al., 2009; Hammer and Bassler, 2003). Although Δ*hapR* mutants produce thicker biofilms compared to the wild-type strain, deletion of the *makD* gene alone does not affect biofilm formation (Hammer and Bassler, 2003). Moreover, analysis of virulence gene expression in hyper-infective *V. cholerae* biofilms reveals no upregulation of any Mak proteins (Gallego-Hernandez et al., 2020). To investigate the role of the MakABE tripartite toxin and the accessory proteins MakCD in the pathogenesis and environmental adaptation of pandemic *V. cholerae* isolates, we analyzed data from previous transcriptomic studies of *V. cholerae* strains under various growth conditions. Notably, we found that the *mak* operon exhibits higher transcriptional activity in marine culture media compared to LB and shows increased expression in minimal growth media such as M63 (https://www.ncbi.nlm.nih.gov/geo/query/acc.cgi?acc=GSE214813; Lang et al., 2021). These findings suggest that the *mak* operon may play a significant role in the bacterium’s ability to adapt and thrive in diverse environmental conditions.

In this study, we demonstrate that MakD and MakC are localized within the bacterial cells and are not secreted, suggesting that these proteins have not evolved to employ a direct toxic effect on host cells. Previously, we showed that *V. cholerae* expressing the MakABE proteins is lethal to both *Caenorhabditis elegans* and zebrafish, whereas a *ΔhapR* mutant, which lacks Mak protein expression, is non-toxic. Furthermore, complementing the *ΔhapR* mutant with either MakC or MakD did not alter nematode survival (Dongre et al., 2018), further supporting that these proteins function as accessory factors without independent toxic effects.

Despite the fact that MakC and MakD share significant sequence similarity (48% sequence identity) and overall structure (0.45 Å RMSD), they exhibit distinct behaviors. When *V. cholerae* was treated with glutaraldehyde and analyzed using MakC or MakD antisera, MakD was largely unaffected, with most of the protein remaining in monomeric form, suggesting limited involvement in protein–protein interactions. On the contrary, analysis with MakC antisera revealed that MakC forms large complexes within the bacterial cell, as evidenced by a reduction in the amount of monomeric protein following treatment, which varied with both time and glutaraldehyde concentration (Figure 5; Supplementary Figures 5, 6). Further investigation is required to determine whether MakC’s interaction partners are components of the flagella or other cytoplasmic proteins, which could shed light on the functional significance of these proteins.

## Data Availability

The datasets presented in this study can be found in online repositories. The names of the repository/repositories and accession number(s) can be found in the article/Supplementary material.
