# Supplementary material for: MakC and MakD are two proteins associated with a tripartite toxin of Vibrio cholerae
Source: Front Microbiol. 2024 Oct 3;15:1457850. doi: 10.3389/fmicb.2024.1457850 (PMC11484084; doi:10.3389/fmicb.2024.1457850)
Supplement: Supplementary file 1 [file Data_Sheet_1.pdf]

MakC and MakD are two proteins associated with a tripartite toxin of *Vibrio cholerae*

Nandita Bodra[1,2], Eric Toh[2,3,4], Aftab Nadeem[2,3], Sun Nyunt Wai [2,3,4] and Karina Persson[1,2]*

[1]Department of Chemistry, Umeå University, 90187, Umeå, Sweden

[2]Umeå Centre for Microbial Research (UCMR), Umeå University, 90187 Umeå, Sweden

[3]Department of Molecular Biology, Umeå University, 90187 Umeå, Sweden

[4]The Laboratory for Molecular Infection Medicine Sweden (MIMS), Umeå University, 90187 Umeå, Sweden

*** Correspondence:**
Corresponding Author: Karina Persson
karina.persson@umu.se

Supplementary figures

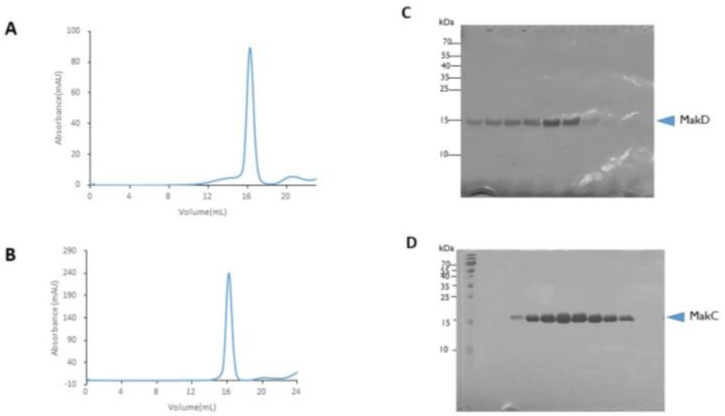

Supplementary figure 1. Purification and analysis of MakC and MakD. Recombinantly expressed MakC and MakD proteins were purified by gel filtration on a Superdex 200 10/300 column (A and B). The obtained protein samples were run on 15 % SDS PAGE gels (C and D).

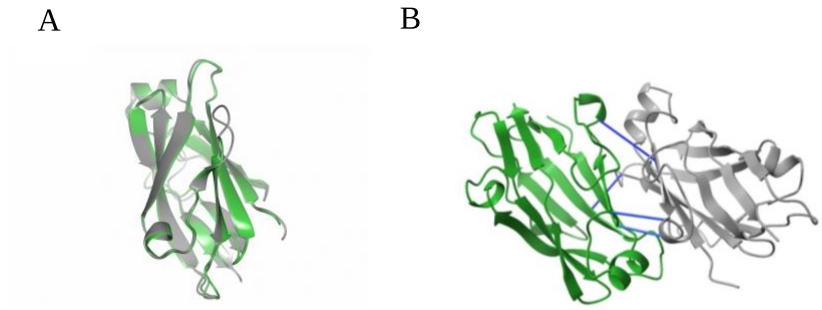

Supplementary figure 2. Comparison of MakC and MakD and putative heterodimer. (A) Crystal structures of MakC (grey) and MakD (green) were superimposed. The structures are structurally similar with only minor differences. The RMSD is 0.45 Å. (B) Prediction of MakC and MakD interactions. With Alphafold modelling MakC (grey) and MakD (green) were predicted to bind each other. Blue line represents the major binding contacts between MakC and MakD residues.

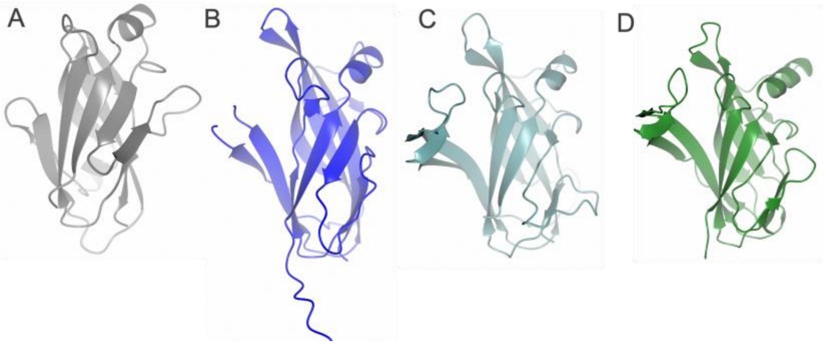

Supplementary figure 3: Comparison of MakD and proteins form the DALI search. The DALI search resulted in hits with low Z-scores A) MakD; B) PDB:4LZK, Inclusion body protein from *B. cenocepacia*. C) PDB:4Q52, uncharacterized protein from *C. pinensis*. D) PDB:4PIB Uncharacterized PixA from *B. thailandensis*.

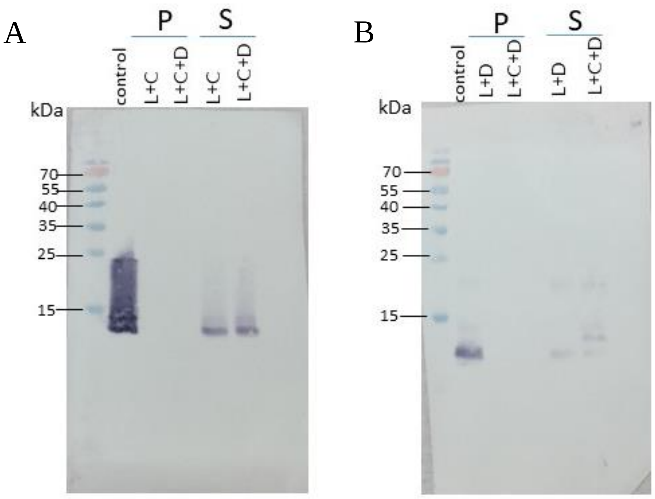

Supplementary figure 4. Liposome pulldown assays: Liposomes prepared from *V. cholerae* lipid extracts were incubated with (A) MakC and (B) MakD and crosslinked with 0.05% glutaraldehyde. The soluble and lipid bound proteins were separated and analyzed with MakC and MakD specific antisera. L: Liposomes, C: MakC, D: MakD, P: Pellet, S: Supernatant.

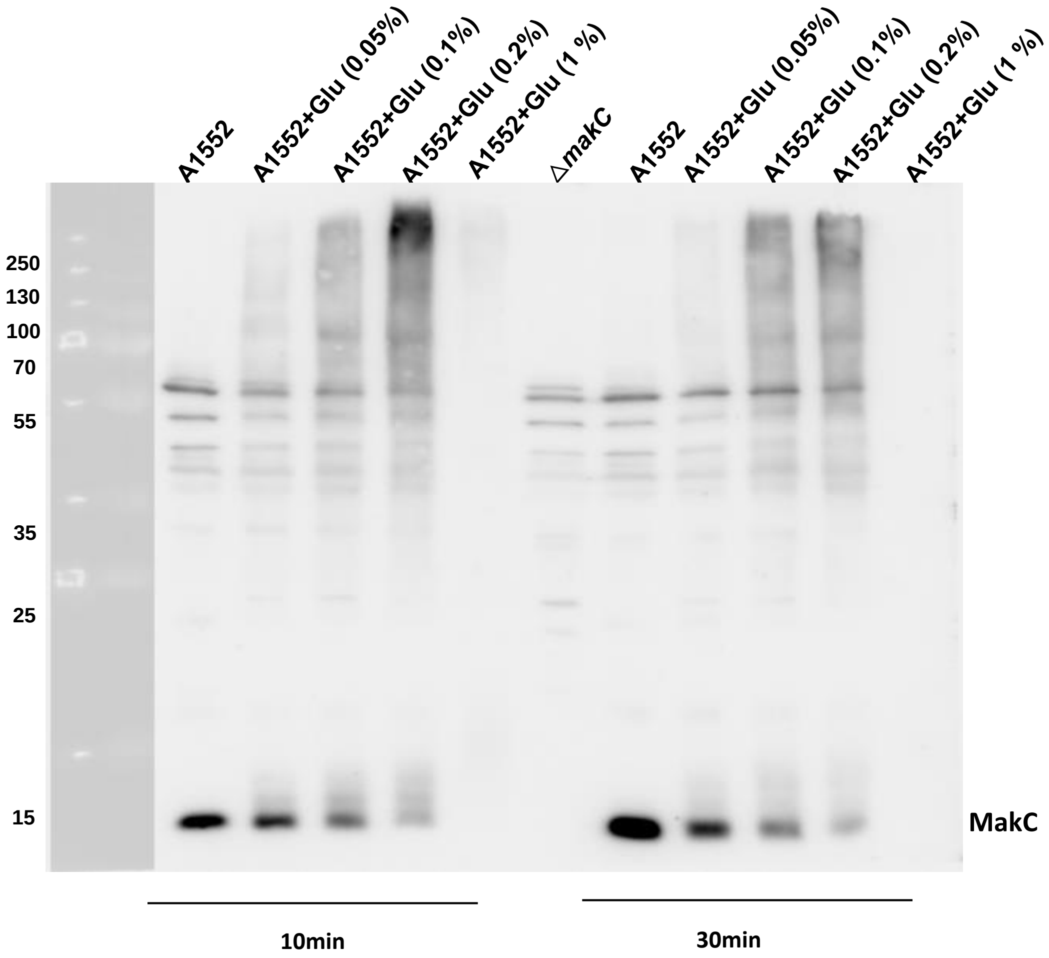

Supplementary figure 5. Analysis of crosslinking of MakC in *V. cholerae* O1 El Tor strain A1552. Untreated WT A1552 (lanes 1 and 7), A1552 Δ*makC* (lane 6) and WT A1552 treated with 0.05%, 0.1%, 0.2% and 1% Glutaraldehyde (lanes 2 -5) for 10 minutes. WT A1552 treated with 0.05%, 0.1%, 0.2% and 1% Glutaraldehyde (lanes 8 -10) for 30 minutes. Analysis was performed with Western immunoblotting using antisera against MakC.

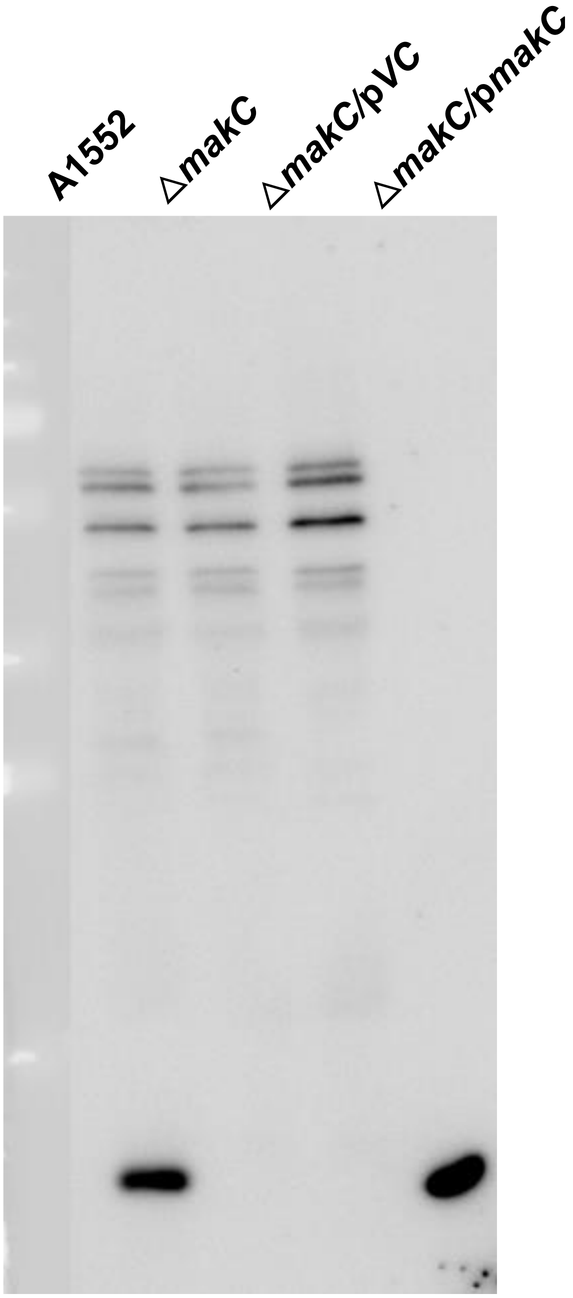

Supplementary figure 6. Immunoblot of *V. cholerae* O1 El Tor strain A1552 with antisera against MakC. WT A1552 (lane 1), A1552 Δ*makC* (lane 2), WT A1552 Δ*makC*+vector control (lane 3) and WT A1552 Δ*makC*+p*makC* (lane 4). Lanes 2 and 3 show the non-specific bands bound by the MakC antisera.

Supplementary Table 1: Bacterial strains and primers used in this study

Strain/ plasmid	Description/relevant characteristics	Reference / Source
Vibrio cholerae A1552: serogroup O1, biotype El Tor, Rif^r		Yildiz et al., 1998(1)
MDS002	A1552Δ*makD*	Dongre et al., 2018(2)
MDS003	A1552Δ*makC*	Dongre et al., 2018(2)
MDS004	A1552Δ*makB*	Dongre et al., 2018(2)
MDS005	A1552Δ*makA*	Dongre et al., 2018(2)
MDC008	A1552Δ*makE*	Nadeem et al. (2021)(3)
MDS006	A1552Δ*makDC*	Dongre et al., 2018(2)
**Primers** (5' to 3')		
MakC-forward	gtaactcatgaaaaaagtagaaatactcatggtc	This study
MakC-reverse	gtaacggtaccttacagtgaaataacgaggaaagg	This study
MakD-forward	gtaactcatgaaaaaaatagaaatacttatcgtg	This study
MakD-reverse	gtaacggtaccttattgaacttcgagataaggtg	This study

Supplementary Table 2: Data processing and refinement statistics

	MakC SeMet	MakD SeMet	MakD native
**Data collection**			
Wavelength	0.9793	0.9793	0.9677
Space group	$C222_1$	$P2_12_12$	$P2_12_12_1$
Cell dimensions			
a, *b*, *c* (Å)	47.0, 98.0, 54.5	107.9, 109.5, 49.0	42.3, 70.9, 185.7
α, β, γ (°)	90.0, 90.0, 90.0	90, 90, 90	90, 90, 90
Resolution (Å) *	36.45-2.05 (2.08-2.05)	49.0-2.12 (2.19-2.12)	46.6-2.02 (2.09-2.02)
R_{merge}	0.177 (1.134)	0.158 (2.627)	0.136 (1.747)
$I / \sigma I$	8.5 (2.0)	11.7 (0.8)	11.1 (1.6)
Completeness (%)	97.1 (100)	100(99.9)	100 (100)
Redundancy	6.8 (7.3)	26.1(25.8)	13.1 (13.9)
CC1/2	0.994 (0.760)	0.997(0.729)	0.998(0.596)
Molecules in a.u.	1	4	4
**Refinement**			

Resolution (Å)	36.48-2.05		38.84-2.02
No. reflections (work/test)	8024 (419)		37603(1415)
R_{work} / R_{free}	0.183 (0.200)		0.188(0.236)
No. atoms			
Protein	1980		3892
Ligand/ion	0		0
Water	47		263
B-factors (Å^2)			
Protein	32.1		53.1
Ligand/ion			
Water	32.3		52.2
R.m.s. deviations			
Bond lengths (Å)	0.0138		0.008
Bond angles (°)	1.99		1.06
PDB code	8RQY		6TCT

1. Yildiz, F. H., and Schoolnik, G. K. (1998) Role of rpoS in stress survival and virulence of Vibrio cholerae. *J Bacteriol* **180**, 773-784
2. Dongre, M., Singh, B., Aung, K. M., Larsson, P., Miftakhova, R., Persson, K., Askarian, F., Johannessen, M., von Hofsten, J., Persson, J. L., Erhardt, M., Tuck, S., Uhlin, B. E., and Wai, S. N. (2018) Flagella-mediated secretion of a novel Vibrio cholerae cytotoxin affecting both vertebrate and invertebrate hosts. *Communications Biology* **1**, 59
3. Nadeem, A., Nagampalli, R., Toh, E., Alam, A., Myint, S. L., Heidler, T. V., Dongre, M., Zlatkov, N., Pace, H., Bano, F., Sjostedt, A., Bally, M., Uhlin, B. E., Wai, S. N., and Persson, K. (2021) A tripartite cytolytic toxin formed by Vibrio cholerae proteins with flagellum-facilitated secretion. *Proc Natl Acad Sci U S A* **118**
